# Transcriptomic Changes Associated with Pregnancy in a Marsupial, the Gray Short-Tailed Opossum *Monodelphis domestica*

**DOI:** 10.1371/journal.pone.0161608

**Published:** 2016-09-06

**Authors:** Victoria Leigh Hansen, Faye Dorothy Schilkey, Robert David Miller

**Affiliations:** 1 Center for Evolutionary and Theoretical Immunology, Department of Biology, University of New Mexico, Albuquerque, New Mexico, United States of America; 2 National Center for Genome Resources/New Mexico IDeA Networks of Biomedical Research Excellence, Santa Fe, New Mexico, United States of America; INRA, FRANCE

## Abstract

Live birth has emerged as a reproductive strategy many times across vertebrate evolution; however, mammals account for the majority of viviparous vertebrates. Marsupials are a mammalian lineage that last shared a common ancestor with eutherians (placental mammals) over 148 million years ago. Marsupials are noted for giving birth to highly altricial young after a short gestation, and represent humans’ most distant viviparous mammalian relatives. Here we ask what insight can be gained into the evolution of viviparity in mammals specifically and vertebrates in general by analyzing the global uterine transcriptome in a marsupial. Transcriptome analyses were performed using NextGen sequencing of uterine RNA samples from the gray short-tailed opossum, *Monodelphis domestica*. Samples were collected from late stage pregnant, virgin, and non-pregnant experienced breeders. Three different algorithms were used to determine differential expression, and results were confirmed by quantitative PCR. Over 900 opossum gene transcripts were found to be significantly more abundant in the pregnant uterus than non-pregnant, and over 1400 less so. Most with increased abundance were genes related to metabolism, immune systems processes, and transport. This is the first study to characterize the transcriptomic differences between pregnant, non-pregnant breeders, and virgin marsupial uteruses and helps to establish a set of pregnancy-associated genes in the opossum. These observations allowed for comparative analyses of the differentially transcribed genes with other mammalian and non-mammalian viviparous species, revealing similarities in pregnancy related gene expression over 300 million years of amniote evolution.

## Introduction

The transition from oviparity to viviparity has occurred independently multiple times in vertebrate evolution. Indeed, most vertebrates including cartilaginous fishes, bony fishes, amphibians, squamate reptiles, and mammals have lineages that transitioned to viviparity from oviparous ancestors [1]. In many viviparous vertebrates the transition away from oviparity involved the evolution of the placenta, which serves as an interface between the maternal system and the conceptus. The placenta is a specialized organ that facilitates the transfer of gases, nutrients, and waste as well as providing a physical barrier to protect the fetus from pathogens [2].

Based on phylogenetic relationships between metatherian (marsupial) and eutherian ("placental") mammals, viviparity appears to have evolved once early in the therian lineage [3–5]. There are three general categories of placental invasiveness in therians. The least invasive is epitheliochorial placentation, which is a superficial apposition of fetal and maternal membranes, where as the most is the hemochorial placenta where fetal trophoblast invades the uterine endometrium and remodels maternal capillaries [6, 7]. The third major type, an intermediary endotheliochorial placenta, also invades into the endometrium but does not directly contact maternal circulation [7]. Eutherians vary from species to species in placental invasiveness [6]. Primates and rodents typically have hemochorial placentation, whereas ruminants like cows and sheep use epitheliochorial placentas [8, 9]. Most branches of eutherians also have species that use endotheliochorial placentation, such as canines and bats [6, 9].

Prior to the widespread use of molecular phylogenetics, most theories of placental evolution were based on assuming a progression from less invasive to more invasive [10]. The current view is that the ancestral eutherian placenta was either hemochorial or endotheliochorial [9, 11–15]. The less invasive epitheliochorial placenta in eutherian species is likely an evolutionary innovation to give the mother greater control over nutrient and gas exchange [14, 15]. There is a correlation between forming an epitheliochorial placenta and giving birth to more precocial offspring in eutherians [14]. Garratt and colleagues [15] hypothesized that this may be due to maternal and fetal interests deviating relatively quickly after birth in many eutherian species with epitheliochorial placentation.

The marsupials are the sister group to eutherians that give birth to relatively altricial young [16]. In contrast to the eutherian placentation trend, marsupials tend to form epitheliochorial-like placentas despite their altricial offspring. Moreover most marsupials use the yolk sac, as opposed to the allantois, to contact the chorion and form the trophoblast that is apposed to maternal membranes [16, 17]. A notable exception is within the family Peramelidae (bandicoots and echymiperas) where an invasive chorioallantoic placenta forms in the last few days before parturition [16, 18].

The morphological changes of the placenta throughout pregnancy have been well characterized in several marsupial species such as *Sminthopsis crassicaudata*, *Macropus eugenii*, and *M*. *domestica* [19–23]. *M*. *domestica* forms an invasive endotheliochorial yolk sac placenta in the last 60 hours prior to birth and, characteristic for marsupials, a permeable shell coat separates the fetal and maternal membranes for the majority of gestation [21]. *M*. *domestica* is a member of the Didelphidae family, considered to be among the least derived marsupial lineage and likely represents the ancestral reproductive characteristics of marsupials [17, 21]. Therefore *M*. *domestica* is potentially an important model for discovering other basal characteristics of reproduction in marsupials and perhaps even therians in general.

More recent analyses of gene expression in reproductive tissues during opossum pregnancy have made important contributions to our understanding of the evolution of the placenta in eutherian mammals [24, 25]. Kin and colleagues described the distribution of important eutherian transcription factors (TFs) in *M*. *domestica* endometrium, determining the ancestral cell type of eutherian endometrial stromal fibroblasts (ESFs) that undergo decidualization [24]. Lynch and colleagues recently revealed that endometrially-expressed transposable elements (TEs), ancient in the eutherian lineage, have played an important role in the evolution of decidual tissues; a tissue type lacking in marsupials [25]. This has demonstrated key adaptations in endometrial tissue that may have enabled the successful extended gestation periods seen in eutherians.

Needed for a greater understanding of the mammalian adaptation to viviparity are analyses of those genes differentially transcribed between pregnant and non-pregnant tissues within a non-eutherian mammal. This may also facilitate the discovery of genes crucial to marsupial pregnancy that may or may not be shared with eutherians or, indeed, other viviparous vertebrates. Presented here is just such a comparison with an analysis of the pregnant and non-pregnant uterine transcriptomes of *M*. *domestica*.

## Results

To investigate changes in gene expression patterns associated with opossum pregnancy, transcriptome analyses were performed on uterine tissues from late stage pregnant (P), virgin (V), and non-pregnant (N) experienced breeders. Three biological replicates were used for each condition. Paired-end reads were generated on the Illumina HiSeq 2000 platform and aligned to the *M*. *domestica* genome. The number of mapped reads per sample ranged from 17.1 to 25.9 million at the high end, with an average of 20.4 million (S1 Table). The numbers of aligned reads per sample were more than sufficient for differential expression analysis with treatment groups containing three biological replicates [26].

**Fig 1:**
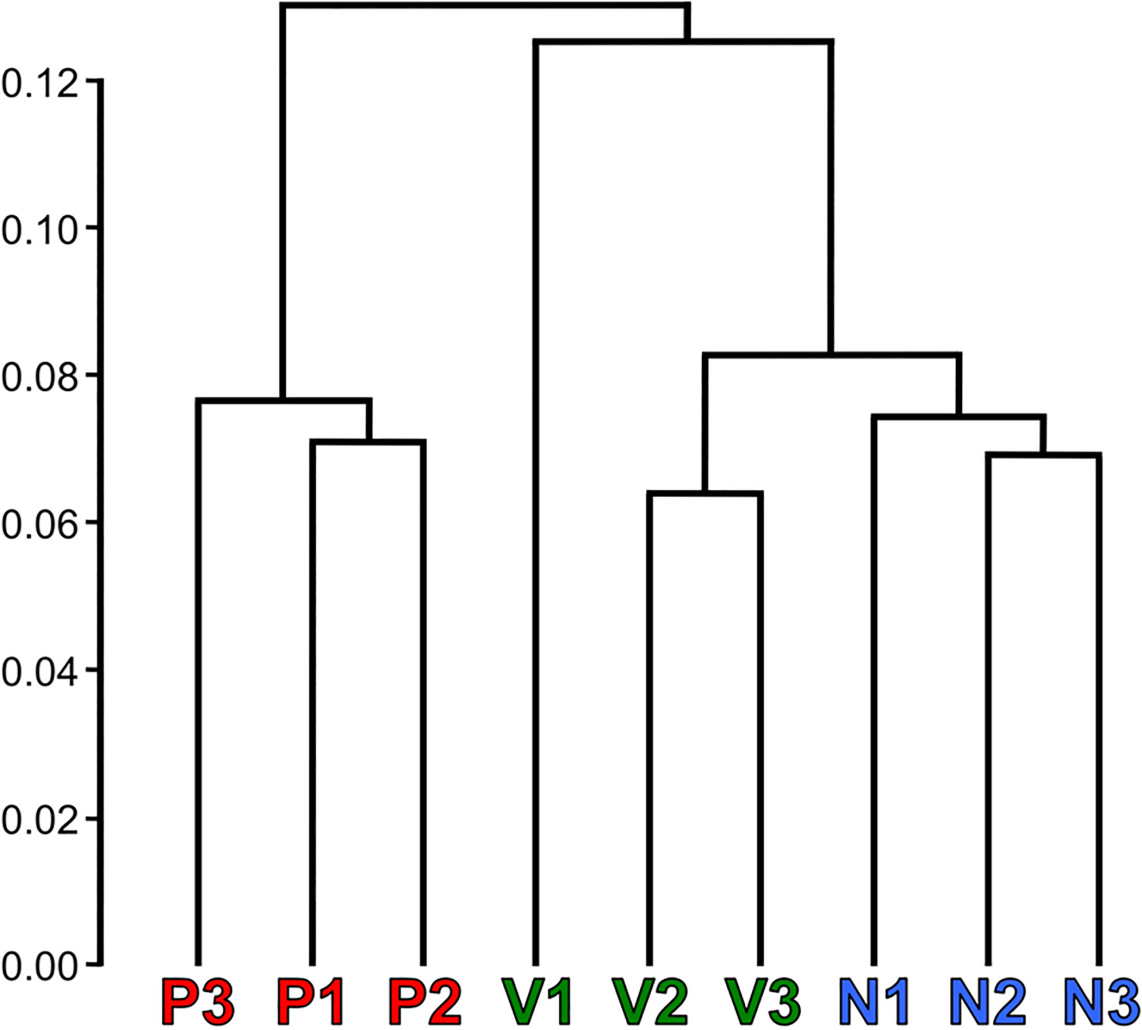
Global transcriptomic similarities between samples. Dendrogram of Jensen-Shannon (JS) distances between transcriptomes of replicates with the pregnant (P), virgin (V), and non-pregnant past breeder (N) transcriptomes.

**Fig 2:**
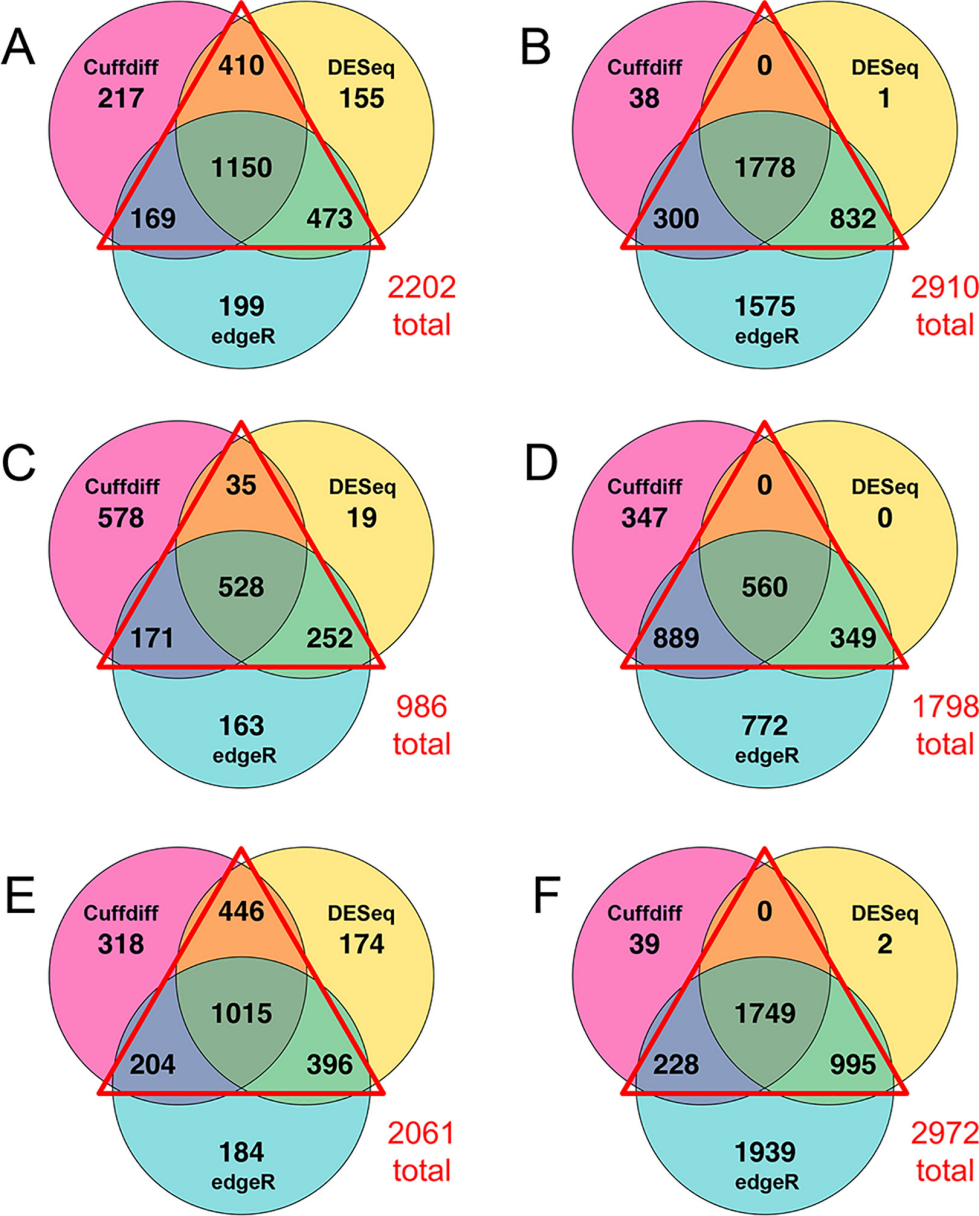
Overlap of genes called as significant in differential expression programs. Venn diagrams of the numbers of gene transcripts found to be significantly more abundant (A, C, and E) or less abundant (B, D, and F) when comparing the dataset from the P group to N (A and B), V (C and D) and V2 & V3 only (E and F). Genes were tested for differential expression according to Cuffdiff (pink), DESeq (yellow), and edgeR (blue). Red triangles denote the cases where at least two out of three programs were in agreement.

Comparing the global transcriptomes individually, the nine samples segregated into pregnant (P) and non-pregnant groups (N and V) based on Jensen-Shannon (JS) distances (Fig 1). The P transcriptomes grouped together and were more similar to each other than they were to any other samples. Of the non-pregnant individuals, all three N grouped together and two of the three V individuals grouped together. Virgin opossum V1 did not group with the other virgin animals, but her uterine transcriptome was more similar to the other non-pregnant animals than to the pregnant animals (Fig 1). For this reason analyses both including and excluding V1 were performed when testing the P group against the V and N groups. No other replicate in any group was dissimilar enough to warrant being separated for further analyses. Three replicates per treatment group appeared to be adequate since they grouped together well overall indicating consistency between biological replicate transcriptomes (Fig 1). The P group was tested for differentially transcribed genes compared to N, all three V, and V2 and V3 alone (V2 & V3) (Fig 2).

The three sample groups, V, N, and P, were compared in a pair-wise manner and independently assessed for differential transcription using Cuffdiff, DESeq, and edgeR. Since these algorithms assess differential expression using different parameters they identified partially, but not fully, overlapping sets of genes (Fig 2). Genes that were identified as being differentially transcribed by at least two of the three programs were used for this study (red triangles in Fig 2).

Transcripts of 2202 genes were significantly more abundant in the P uterus compared to N (Fig 2A), whereas 2910 genes were significantly less (Fig 2B). When the P samples were compared to all three V only 986 gene transcripts increased in abundance (Fig 2C). However when V1 was excluded this number was 2061 (Fig 2E), more similar to the comparison with N (Fig 2A). There were 1798 and 2972 decreased gene transcripts in the same comparisons, respectively (Fig 2D and 2F). Therefore, including the outlier V1 suppressed the number of differentially genes. Its exclusion made the V group more similar to N in numbers of differentially transcribed genes.

### Validation of differential gene transcript abundance

**Fig 3:**
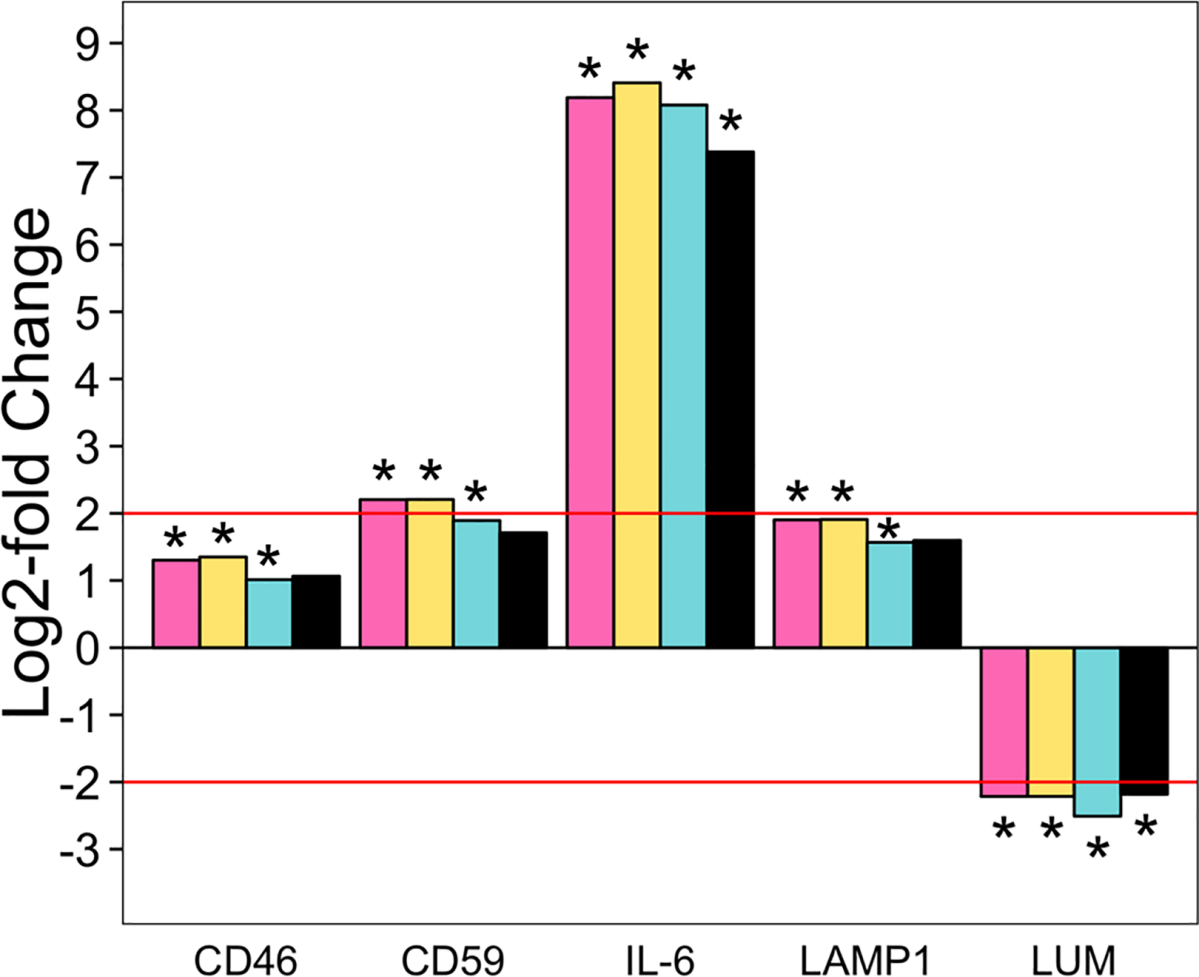
Gene transcription levels in P vs N according to differential expression programs compared to qPCR data. Log2-fold changes in the transcription of membrane cofactor protein (CD46), MAC-inhibitory protein (CD59), interleukin 6 (IL-6), lysosomal-associated membrane protein 1 (LAMP1), and lumican (LUM) in the pregnant group compared to the non-pregnant past breeder group. Log2-fold changes were according to Cuffdiff (pink bars), DESeq (yellow bars), edgeR (blue bars), and qPCR using the Vandesompele method (black bars). Red line indicates the threshold of log2-fold change needed for significance according to the Vandesompele method of relative quantification of qPCR data. * Adjusted p < 0.05 for differential expression programs or >2 log2-fold change for qPCR.

To validate the Illumina data, we developed quantitative PCR (qPCR) assays for a subset of genes that were differentially represented in the different groups. These were *membrane cofactor protein* (*CD46*), *MAC-inhibitory protein* (*CD59*), *interleukin-6* (*IL6*), *lysosomal-associated membrane protein 1* (*LAMP1*), and *lumican* (*LUM*). The direction of log2-fold change of gene expression in P compared to N samples in the Illumina datasets was recapitulated by the qPCR (Fig 3). The same was found in a P vs V comparison. These genes were not significantly differentially expressed in V compared to the N group in either Illumina or by qPCR (S1 Fig).

### Pregnancy-associated genes in the opossum

**Fig 4:**
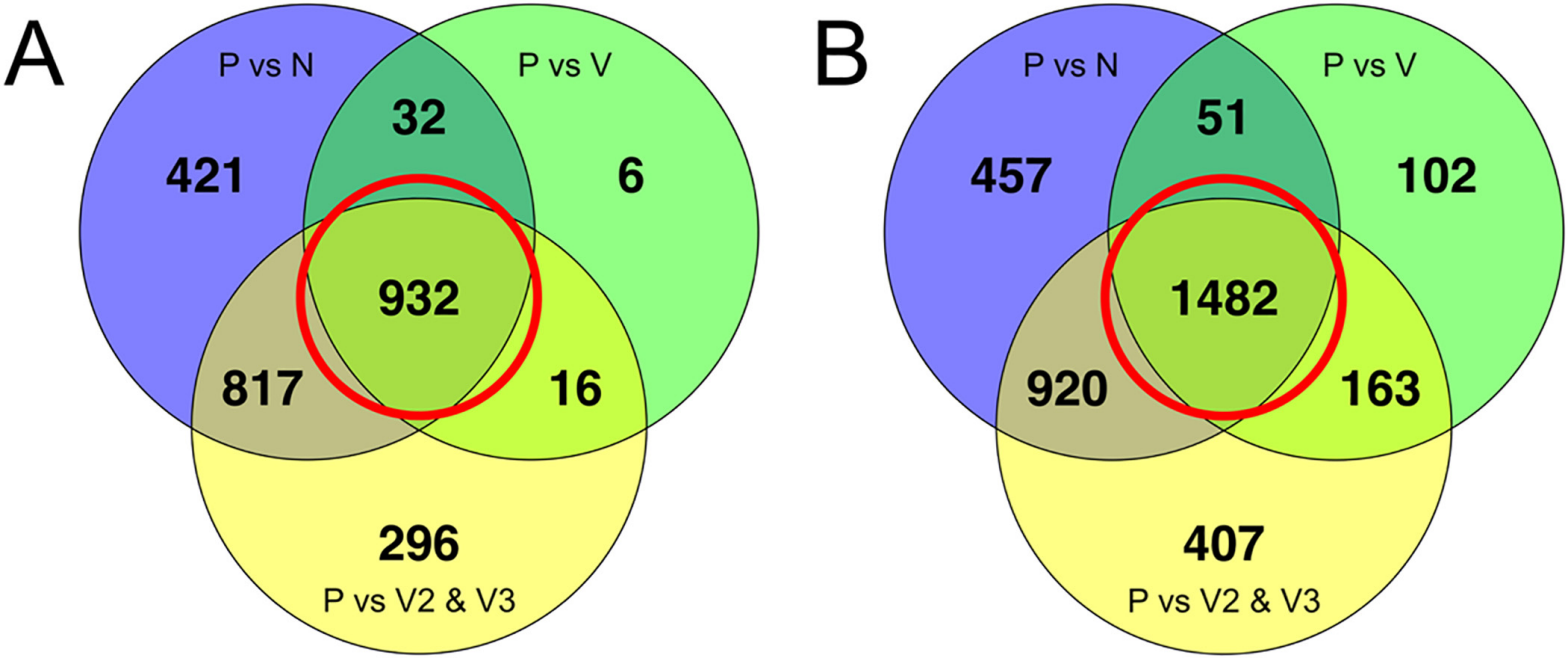
Pregnancy-associated gene sets. Venn diagrams describing the intersection of genes that were considered differentially transcribed in comparisons of the P group to the N, V, and V2 & V3 control groups. The genes that were differentially transcribed in the P group compared to all three control groups (red circles) were considered to be differentially transcribed at a high level of confidence for this study.

To determine gene sets that were explicitly differentially transcribed during pregnancy, the intersections of differentially transcribed genes in the P set compared to both non-pregnant sets (N and V) were identified. Such genes should represent a minimal, high confidence “pregnancy-associated” set for the opossum. In the intersections of all three comparisons 932 genes were significantly more abundant and 1482 genes were less abundant in the P group (Fig 4). These increased and decreased differentially transcribed pregnancy-associated genes are listed in S2 and S3 Tables. Gene Ontology (GO) analyses of the pregnancy defining genes revealed 18 GO terms were overrepresented and 6 GO terms were underrepresented (Table 1). In the pregnancy-associated genes with decreased transcription during pregnancy, 25 GO terms were overrepresented and two were underrepresented (Table 2). The most overrepresented GO term in the increased in pregnant gene set was ion transport (p = 9.86E-06) (Table 1); the most overrepresented in the decreased in pregnant gene set was ectoderm development (p = 2.23E-09) (Table 2).

**Table 1:** Overrepresented and underrepresented Biological Process GO terms in the increased transcription during pregnancy gene set.

**Overrepresented GO-Slim Biological Process**	**P-value**
ion transport	9.86E-06
immune system process	9.15E-05
proteolysis	1.08E-04
cellular amino acid metabolic process	2.12E-04
amino acid transport	3.04E-04
cation transport	4.99E-04
lipid metabolic process	7.04E-04
cell-matrix adhesion	1.08E-03
blood coagulation	1.12E-03
extracellular transport	1.40E-03
cell-cell adhesion	3.53E-03
angiogenesis	4.64E-03
response to external stimulus	7.30E-03
transport	1.14E-02
response to stimulus	1.52E-02
system development	1.61E-02
localization	2.15E-02
blood circulation	2.42E-02
**Underrepresented GO-Slim Biological Process**	**P-value**
nucleobase-containing compound metabolic process	1.98E-06
RNA metabolic process	4.21E-05
cell cycle	2.90E-03
translation	4.79E-03
DNA metabolic process	1.34E-02
mRNA processing	1.42E-02

**Table 2:** Overrepresented and underrepresented Biological Process GO terms in the decreased transcription during pregnancy gene set.

**Overrepresented GO-Slim Biological Process**	**P-value**
cellular process	2.92E-14
ectoderm development	2.23E-09
nervous system development	5.17E-09
multicellular organismal process	9.91E-09
single-multicellular organism process	9.91E-09
chromosome segregation	3.10E-08
cell-cell adhesion	9.10E-08
developmental process	2.02E-07
system process	2.76E-07
cell adhesion	4.81E-07
biological adhesion	4.81E-07
system development	5.78E-07
cell cycle	2.31E-06
neurological system process	4.95E-06
mitosis	4.59E-05
cellular component organization	1.68E-04
cell communication	4.66E-04
cellular component organization or biogenesis	6.41E-04
muscle organ development	1.35E-03
cell-cell signaling	1.73E-03
cellular component morphogenesis	8.77E-03
mesoderm development	1.18E-02
cellular component movement	1.81E-02
cytokinesis	2.99E-02
muscle contraction	3.23E-02
**Underrepresented GO-Slim Biological Process**	**P-value**
primary metabolic process	2.47E-03
metabolic process	8.20E-03

### Differential transcript abundance between pre- vs. post-estrus

There is evidence that marsupial uterine differentiation is not completed until the first estrus occurs [16, 27]. The data sets generated for this study allow for investigation of transcriptomic differences between the N and V groups that might be linked to this differentiation. The V and N samples were transcriptomically similar to each other when compared to the P group (Fig 1). In order to determine the genes that might be linked to uterine differentiation, the pre-estrus V dataset was compared to the post-estrus N dataset. The intersections of N vs V and N vs V2 & V3 were examined and 8 genes were significantly more abundant and 4 were less abundant in V relative to N, respectively (Table 3).

**Table 3:** Significantly differentially transcribed genes in the virgin group as compared to the non-pregnant past breeder group.

Expression in V Group	Gene Symbol	Ensembl Gene ID	Gene Description
Increased	SLC6A15	ENSMODG00000020492	Solute carrier family 6 (neutral amino acid transporter), member 15
Increased	TFAP2C	ENSMODG00000016445	Transcription factor AP-2 gamma (activating enhancer binding protein 2 gamma
Increased	GPR115	ENSMODG00000018861	G protein-coupled receptor 115
Increased	ARG1	ENSMODG00000017584	Arginase 1
Increased	ZAR1	ENSMODG00000020634	Zygote arrest 1
Increased	ACTC1	ENSMODG00000001384	Actin, alpha, cardiac muscle 1
Increased	KRT13	ENSMODG00000014608	Keratin 13
Increased	RAB3B	ENSMODG00000000791	RAB3B, member RAS oncogene family
Decreased	NTSR1	ENSMODG00000016798	Neurotensin receptor 1 (high affinity)
Decreased	IGK^a^	ENSMODG00000009815	Ig kappa chain constant domain
Decreased	IGH^a^	ENSMODG00000029124	IgG heavy chain constant domain
Decreased	IGJ	ENSMODG00000019083	Immunoglobulin J polypeptide, linker protein for immunoglobulin alpha and mu polypeptides

^a^ Not annotated for *M*. *domestica* in Ensembl; BLASTN of gene sequence

### Conservation of genes regulated during pregnancy

Transcriptomic changes in *M*. *domestica* uterus during pregnancy were compared to previously published uterine transcriptome studies in pig (*Sus scrofa*), skink (*Chalcides ocellatus*), and seahorse (*Hippocampus abdominalis*) [28–30]. For this meta-analysis, “top-100” gene sets were identified. The top-100 significantly increased genes in opossum pregnancy are the 100 genes with the greatest transcript abundance among the 932 increased in P (Fig 4A). These were then compared to the published analyzed differentially expressed gene sets for the pig, skink, and seahorse [28–30]. Fifty-five of the top 100 gene transcripts most abundant during pregnancy for the opossum were identified as also increased in pregnant skink (Table 4). Only 11 and 1 of the selected top 100 opossum genes were identified as increased in pregnant pig and seahorse, respectively (Table 4). Of the 1482 genes identified as being decreased in pregnant (Fig 4B), the top 100 is that subset that had the greatest abundance of transcripts in the opossum N samples. Forty-three of these genes were also identified as significantly decreased in skink pregnancy (Table 5). Only 8 of these were identified in the pig pregnancy and none in the seahorse analyses (Table 5).

**Table 4:** Comparison of “top 100” genes with significantly higher transcription during pregnancy than a non-pregnant state for opossum (*M*. *domestica*), skink (*C*. *ocellatus*), pig (*S*. *scrofa*), and seahorse (*H*. *abdominalis*).

Gene Symbol	Gene Description	Average Opossum Count in Pregnant	Average Opossum Log2-fold Change	Skink Log2-fold Change^a^	Pig Log2-fold Change^b^	Seahorse Log2-fold Change^c^
RBP4	retinol binding protein 4 plasma	144035	8.14	9.04	0.8	N/A
SOD3	superoxide dismutase 3 extracellular	136902	7.53	1.64	N/A	N/A
LAMC1	laminin gamma 1	90448	3.47	0.93	N/A	N/A
MFGE8	milk fat globule-EGF factor 8 protein	68679	2.34	0.96	NA	N/A
VEGFA	vascular endothelial growth factor A	57448	5.16	1.68	N/A	N/A
FN1	fibronectin 1	56309	2.86	2.91	0.9	N/A
OAT	ornithine aminotransferase	55577	3.91	1.25	N/A	N/A
TC2N	tandem C2 domains nuclear	46083	4.62	4.22	N/A	N/A
FUCA2	fucosidase alpha-L- 2 plasma	45619	5.74	8.13	N/A	N/A
HPGD	hydroxyprostaglandin dehydrogenase 15-(NAD)	44649	3.00	4.46	N/A	N/A
APOA4	apolipoprotein A-IV	35713	11.53	2.33	N/A	N/A
FBLN1	fibulin 1	35636	5.01	0.53	N/A	N/A
APOA1	apolipoprotein A-I	34757	10.18	6.19	N/A	6.7
CD9	Tetraspanin	33256	2.12	1.13	N/A	N/A
GLUL	glutamate-ammonia ligase	31378	2.42	4.47	N/A	N/A
BSG	basigin	30580	2.41	2	N/A	N/A
CTGF	connective tissue growth factor	27365	4.11	2.09	-1.5	N/A
S100A6	S100 calcium binding protein A6	25414	9.61	1.43	N/A	N/A
UGP2^d^	UDP-glucose pyrophosphorylase 2	23790	4.07	2.77	0.8	N/A
CA15^d^	carbonic anhydrase 15-like)	22857	7.98	4	N/A	N/A
SGK1	serum/glucocorticoid regulated kinase 1	21241	4.43	3.79	0.9	N/A
CLU	clusterin	18230	1.88	1.88	N/A	N/A
LAMP1	lysosomal-associated membrane protein 1	17351	1.96	0.6	N/A	N/A
SLC39A9	solute carrier family 39 member 9	16924	2.04	1.57	N/A	N/A
KRT18	keratin 18	16287	2.98	2.6	-1.6	N/A
SLC41A2	solute carrier family 41 member 2	16039	4.65	0.71	N/A	N/A
CTSL1-like^d^	cathepsin L1-like	15665	3.12	9.8	N/A	N/A
SLC16A5	solute carrier family 16 member 5	15270	5.02	1.86	N/A	N/A
LGMN	legumain	14692	1.69	7.8	N/A	N/A
SPINT2	serine peptidase inhibitor Kunitz type 2	14629	2.29	2.39	N/A	N/A
ABCA2	ATP-binding cassette sub-family A member 2	14471	1.37	0.45	N/A	N/A
HEXB	hexosaminidase B	13839	2.82	7.09	0.7	N/A
PCK1	phosphoenolpyruvate carboxykinase 1	13158	4.54	7.76	7.7	N/A
GLRX	glutaredoxin	12803	2.58	2.46	1.5	N/A
HIF1A	hypoxia inducible factor 1 alpha subunit	12033	1.43	1.13	N/A	N/A
EDEM3	ER degradation enhancer mannosidase alpha-like 3	11608	3.14	1.18	N/A	N/A
CTSA	cathepsin A	11252	2.72	3.49	N/A	N/A
EZR^d^	ezrin	11085	2.00	1.67	N/A	N/A
ATP6V0A4	ATPase H+ transporting lysosomal V0 subunit a4	10973	5.30	3.53	N/A	N/A
TACSTD2	tumor-associated calcium signal transducer 2	10907	2.53	3.21	N/A	N/A
F3	coagulation factor III	10317	3.88	0.56	N/A	N/A
FERMT1	fermitin family member 1	9972	3.47	1.2	N/A	N/A
GPX3	glutathione peroxidase 3	9890	2.81	7.01	1.9	N/A
FGA	fibrinogen alpha chain	9829	9.29	4.25	N/A	N/A
MDM2	MDM2 oncogene E3 ubiquitin protein ligase	9771	2.56	0.49	N/A	N/A
DPP4	dipeptidyl-peptidase 4	9723	2.94	5.17	N/A	N/A
RHOU	ras homolog family member U	9413	2.59	2.55	N/A	N/A
CD164	CD164 molecule sialomucin	9376	1.72	0.97	N/A	N/A
PRCP	prolylcarboxypeptidase (angiotensinase C)	9109	4.63	4.81	1.8	N/A
CCNG2	cyclin G2	9076	2.77	0.51	N/A	N/A
CDO1	cysteine dioxygenase type 1	8886	5.03	2.98	-1.9	N/A
ENTPD5	ectonucleoside triphosphate diphosphohydrolase 5	8853	2.53	2.33	N/A	N/A
HEBP1	heme binding protein 1	8800	3.28	1.71	N/A	N/A
LIMA1	LIM domain and actin binding 1	8781	1.39	1.72	N/A	N/A
DAG1	dystroglycan 1	8257	1.71	0.96	N/A	-1.1
AURKAIP1^d^	aurora kinase A interacting protein 1	175222	10.98	Not DE	N/A	N/A
SLC16A1	solute carrier family 16 member 1	90219	5.11	Not DE	3.5	N/A
GJA1	gap junction protein alpha 1	28936	2.98	Not DE	N/A	N/A
CLSTN1	calsyntenin 1	25541	3.10	Not DE	N/A	N/A
SEC62	SEC62 homolog	22111	1.58	Not DE	N/A	N/A
SAT1	spermidine/spermine N1-acetyltransferase 1	20683	3.02	Not DE	N/A	N/A
SDC4	syndecan 4	17065	2.21	Not DE	N/A	N/A
CCNG1	cyclin G1	15842	1.56	Not DE	N/A	N/A
GNAI1	G protein alpha inhibiting activity polypeptide 1	10469	2.27	Not DE	N/A	N/A
ERBB3	Erb-B2 receptor tyrosine kinase 3	8182	2.20	Not DE	1.2	N/A
TIMP3	TIMP metallopeptidase inhibitor 3	423559	5.04	-2.51	N/A	N/A
IGFBP3	insulin-like growth factor binding protein 3	365182	5.62	-1.4	N/A	N/A
PLAT	plasminogen activator tissue	58851	3.70	-0.61	N/A	N/A
SLCO2A1	solute carrier organic anion transporter family member 2A1	53304	6.80	-0.56	N/A	-1.6
PTGS2	prostaglandin-endoperoxide synthase 2	52902	6.44	-4.64	-1.7	N/A
SRD5A2	steroid-5-alpha-reductase alpha polypeptide 2	40909	7.54	-4.03	N/A	N/A
UCK2	uridine-cytidine kinase 2	34080	4.08	-0.72	N/A	N/A
TSC22D1	TSC22 domain family member 1	21251	2.85	-1.46	N/A	N/A
WFDC2	WAP four-disulfide core domain 2	18812	1.88	-0.91	N/A	N/A
SLC39A8	solute carrier family 39 member 8	18386	3.51	-1.83	1.2	N/A
PROM1	prominin 1	18127	4.68	-1.7	N/A	N/A
RNASEL	ribonuclease L	14498	2.67	-2.12	N/A	N/A
MET	met proto-oncogene	12403	3.11	-0.7	N/A	N/A
APOB^d^	apolipoprotein B	8530	9.77	-3.32	N/A	N/A
ULK2	unc-51 like autophagy activating kinase 2	8521	1.81	-0.63	N/A	N/A
SERPINA1	serpin peptidase inhibitor clade A member 1	124987	8.30	N/A	N/A	N/A
STC1	stanniocalcin 1	93090	9.48	N/A	N/A	N/A
HBE1-like^d^	hemoglobin subunit epsilon-like	43294	7.45	N/A	N/A	N/A
AFP	alpha-fetoprotein	42044	10.50	N/A	N/A	N/A
GABRP	gamma-aminobutyric acid A receptor pi	35781	2.29	N/A	N/A	N/A
PIGR	polymeric immunoglobulin receptor	28437	4.90	N/A	N/A	N/A
CSTL1-like^d^	cystatin-like	26624	3.46	N/A	N/A	N/A
SLC34A2-like^d^	sodium-dependent phosphate transport protein 2B-like	13004	5.03	N/A	N/A	N/A
ESM1	endothelial cell-specific molecule 1	12730	6.66	N/A	N/A	N/A
MDFIC	MyoD family inhibitor domain containing	12031	2.08	N/A	N/A	N/A
RBBP9	retinoblastoma binding protein 9	10542	5.81	N/A	N/A	N/A
IL6	interleukin 6	10525	8.59	N/A	N/A	N/A
FXYD3^d^	FXYD domain containing ion transport regulator 3	10495	3.86	N/A	N/A	N/A
SLC2A1	solute carrier family 2 member 1	10334	3.24	N/A	3.3	N/A
SUCO	SUN domain containing ossification factor	10163	2.08	N/A	N/A	N/A
OXTR	oxytocin receptor	9637	4.46	N/A	N/A	N/A
PC	pyruvate carboxylase	9626	2.40	N/A	N/A	N/A
IL1R2	interleukin 1 receptor type II	9248	4.28	N/A	4.5	N/A
CLIC6	chloride intracellular channel 6	9202	4.03	N/A	N/A	N/A
FAM213A	family with sequence similarity 213 member A	8574	4.33	N/A	N/A	N/A

^a^ Data pulled from Brandley et al. 2012

^b^ Data pulled from Samborski et al. 2013

^c^ Data pulled from Whittington et al. 2015

^d^ Not annotated for *M*. *domestica* in Ensembl; BLASTN of gene sequence

**Table 5:** Comparison of “top 100” genes with significantly lower transcription during pregnancy than a non-pregnant state for opossum (*M*. *domestica*), skink (*C*. *ocellatus*), pig (*S*. *scrofa*), and seahorse (*H*. *abdominalis*).

Gene Symbol	Gene Description	Average Opossum Count in Non-pregnant	Average Opossum Log2-fold Change	Skink Log2-fold Change^a^	Pig Log2-fold Change^b^	Seahorse Log2-fold Change^c^
MYH11	myosin heavy chain 11 smooth muscle	146637	-2.06	-2.3	N/A	N/A
RPL3	ribosomal protein L3	60395	-1.79	-0.11	N/A	N/A
MYLK	myosin light chain kinase	39309	-1.73	-2.52	N/A	N/A
RPS24	ribosomal protein S24	30918	-1.35	-1.38	N/A	N/A
DPT	dermatopontin	23739	-2.58	-4.91	N/A	N/A
RPL23	ribosomal protein L23	23312	-1.29	-0.97	N/A	N/A
SMOC2	SPARC related modular calcium binding 2	22716	-3.88	-2.52	N/A	N/A
IGFBP5	insulin-like growth factor binding protein 5	21266	-3.18	-4.11	N/A	N/A
RPS8	ribosomal protein S8	20773	-1.33	-1.67	N/A	N/A
EEF1B2	eukaryotic translation elongation factor 1 beta 2	20740	-1.70	-1.02	N/A	N/A
RPL35	ribosomal protein L35	20346	-1.80	-0.48	N/A	N/A
RPS20	ribosomal protein S20	20268	-1.82	-0.53	N/A	N/A
RPL22	ribosomal protein L22	20209	-1.72	-1.02	N/A	N/A
RGS5	regulator of G-protein signaling 5	19385	-2.50	-3.33	N/A	N/A
VCAN	versican	18616	-2.19	-2	N/A	N/A
RPS27	ribosomal protein S27	18449	-1.23	-0.43	N/A	N/A
DST	dystonin	17957	-2.22	-2.43	N/A	N/A
PTRF	polymerase I and transcript release factor	17692	-1.70	-0.99	N/A	N/A
FGFR2	fibroblast growth factor receptor 2	13337	-2.12	-1.33	N/A	N/A
PGR	progesterone receptor	12650	-2.77	-3.05	N/A	N/A
RPL30	ribosomal protein L30	11864	-1.28	-1.94	N/A	N/A
DDX50	DEAD (Asp-Glu-Ala-Asp) box polypeptide 50	11747	-1.92	-1.53	N/A	N/A
PPP1R12B	protein phosphatase 1 regulatory subunit 12B	11508	-2.37	-1.93	N/A	N/A
CD55	decay accelerating factor for complement	10820	-4.27	-1.23	N/A	N/A
LMNB2	lamin B2	9510	-1.41	-1.04	N/A	N/A
HNRNPA1^d^	heterogeneous nuclear ribonucleoprotein A1	8817	-2.49	-1.53	N/A	N/A
PDGFRA	platelet-derived growth factor receptor alpha polypeptide	8580	-1.94	-1.15	N/A	N/A
MYH10	myosin heavy chain 10 non-muscle	7912	-2.43	-1.01	N/A	N/A
ALCAM	activated leukocyte cell adhesion molecule	7551	-1.88	-1.18	0.8	N/A
LAMA4	laminin alpha 4	7452	-2.36	-2.6	N/A	N/A
CCDC135	coiled-coil domain containing 135	7242	-3.56	-1.63	N/A	N/A
NR2F2	nuclear receptor subfamily 2 group F member 2	6722	-1.68	-2.12	N/A	N/A
EMILIN1	elastin microfibril interfacer 1	6590	-1.77	-1.62	-0.7	N/A
PPP1R12A	protein phosphatase 1 regulatory subunit 12A	6479	-1.72	-1.27	N/A	1.7
TPPP3	tubulin polymerization-promoting protein family member 3	6362	-2.21	-0.66	N/A	N/A
SYNM	synemin intermediate filament protein	6298	-1.86	-1.7	N/A	N/A
EDNRA	endothelin receptor type A	6105	-3.05	-1.96	N/A	N/A
LTBP1	latent transforming growth factor beta binding protein 1	6017	-1.61	-1.6	N/A	N/A
EFEMP1	EGF containing fibulin-like extracellular matrix protein 1	6003	-1.89	-2.77	-0.8	N/A
SEMA3C	semaphorin 3C	5929	-2.06	-0.74	-1.3	N/A
MDN1	midasin homolog (yeast)	5918	-1.22	-2.92	N/A	N/A
HMGB2	high mobility group box 2	5717	-1.81	-1.28	1	N/A
LBR	lamin B receptor	5421	-1.94	-2.78	N/A	N/A
COL15A1	collagen type XV alpha 1	29091	-4.40	Not DE	N/A	N/A
SERPINE2	serpin peptidase inhibitor clade E member 2	21379	-2.17	Not DE	1.6	1.6
SPOCK3	testican 3	12675	-6.37	Not DE	N/A	N/A
WDR52	WD repeat domain 52	12099	-3.03	Not DE	N/A	N/A
CCDC40	coiled-coil domain containing 40	10227	-3.37	Not DE	N/A	N/A
OLFM4	olfactomedin 4	10012	-2.97	Not DE	N/A	N/A
SYNE1	spectrin repeat containing nuclear envelope 1	9834	-2.62	Not DE	N/A	N/A
ZAN	zonadhesin	8676	-5.96	Not DE	N/A	N/A
SULF1	sulfatase 1	7471	-2.01	Not DE	N/A	N/A
WDR78	WD repeat domain 78	7202	-3.58	Not DE	N/A	N/A
PDLIM3	PDZ and LIM domain 3	7000	-2.16	Not DE	-3	N/A
GPAM	glycerol-3-phosphate acyltransferase mitochondrial	6496	-1.60	Not DE	N/A	N/A
ADAMTS2	ADAM metallopeptidase with thrombospondin type 1 motif 2	6288	-2.07	Not DE	N/A	N/A
DNAH9	dynein axonemal heavy chain 9	6100	-2.72	Not DE	N/A	N/A
HTRA3	HtrA serine peptidase 3	5795	-3.19	Not DE	N/A	N/A
CCDC39	coiled-coil domain containing 39	5639	-3.13	Not DE	N/A	N/A
TEKT2	tektin 2	5538	-4.33	Not DE	N/A	N/A
KIF9	kinesin family member 9	5504	-3.09	Not DE	N/A	N/A
MAK	male germ cell-associated kinase	5409	-2.23	Not DE	N/A	N/A
GSTA4	glutathione S-transferase alpha 4	5073	-6.82	Not DE	N/A	N/A
GSN	gelsolin	27062	-1.64	2.53	N/A	N/A
ZWILCH	zwilch kinetochore protein	18376	-1.73	0.86	N/A	N/A
EIF3D	eukaryotic translation initiation factor 3 subunit D	16024	-1.24	0.24	N/A	N/A
MSLN	mesothelin	14443	-4.24	6.88	-2.9	N/A
ANXA6	annexin A6	10093	-1.57	1.35	N/A	N/A
ENPP2	ectonucleotide pyrophosphatase/phosphodiesterase 2	8322	-2.93	8.52	N/A	N/A
PCOLCE	procollagen C-endopeptidase enhancer	8165	-2.65	3.07	N/A	N/A
TOP2A	topoisomerase II alpha	8162	-4.78	5.36	N/A	N/A
TNFRSF19	tumor necrosis factor receptor superfamily member 19	6330	-3.23	4.17	N/A	N/A
CALCOCO2	calcium binding and coiled-coil domain 2	6299	-1.65	1.16	N/A	N/A
LDHB	L-lactate dehydrogenase B chain	6010	-3.21	0.7	N/A	N/A
DNAH2	dynein axonemal heavy chain 2	5401	-4.42	1.62	N/A	N/A
DMBT1	deleted in malignant brain tumors 1	5398	-3.91	9.5	N/A	N/A
MFSD4	major facilitator superfamily domain containing 4	5167	-5.69	5.17	-1.4	N/A
KRT7	keratin 7	5166	-1.73	1.86	N/A	N/A
EIF5A2	eukaryotic translation initiation factor 5A2	20527	-1.89	N/A	N/A	N/A
CCDC78	coiled-coil domain containing 78	18291	-3.31	N/A	N/A	N/A
NPTX2	neuronal pentraxin II	14803	-7.71	N/A	N/A	N/A
COL14A1	collagen type XIV alpha 1	13659	-4.09	N/A	N/A	N/A
CCDC108	coiled-coil domain containing 108	9196	-4.18	N/A	N/A	N/A
CCDC141	coiled-coil domain containing 141	9031	-2.23	N/A	N/A	N/A
FILIP1L	filamin A interacting protein 1-like	8940	-2.14	N/A	N/A	N/A
COL11A2	collagen type XI alpha 2	8803	-2.17	N/A	N/A	N/A
CIRBP	cold inducible RNA binding protein	8169	-1.30	N/A	N/A	N/A
ARMC3	armadillo repeat containing 3	7039	-4.05	N/A	N/A	N/A
VTCN1	V-set domain containing T cell activation inhibitor 1	6889	-3.22	N/A	-3	N/A
F10	coagulation factor X	6794	-2.32	N/A	N/A	N/A
C15orf26	chromosome 15 open reading frame 26	6624	-4.31	N/A	N/A	N/A
C1orf87	chromosome 1 open reading frame 87	6467	-3.59	N/A	N/A	N/A
unknown	Uncharacterized protein ENSMODG00000023422	6271	-2.56	N/A	N/A	N/A
CA3	carbonic anhydrase III	6219	-3.87	N/A	4	N/A
CKAP5	cytoskeleton associated protein 5	6212	-1.21	N/A	N/A	N/A
MBL2	mannose-binding lectin 2	6109	-6.70	N/A	N/A	N/A
MXRA5	matrix-remodelling associated 5	5872	-3.67	N/A	N/A	N/A
TWSG1	twisted gastrulation BMP signaling modulator 1	5738	-2.08	N/A	N/A	N/A
HDAC2	histone deacetylase 2	5627	-1.24	N/A	N/A	N/A
DPYSL3	dihydropyrimidinase-like 3	5169	-2.07	N/A	-0.9	N/A

^a^ Data pulled from Brandley et al. 2012

^b^ Data pulled from Samborski et al. 2013

^c^ Data pulled from Whittington et al. 2015

^d^ Not annotated for *M*. *domestica* in Ensembl; BLASTN of gene sequence

## Discussion

Pregnancy involves substantial tissue remodeling and physiological changes. This study was aimed at investigating the changes that occur at the level of uterine gene transcription in a model marsupial species. Marsupials are a distinct lineage of mammals noteworthy for their short gestation times, relative immaturity at birth, and minimal placentation [16]. Viviparity likely has a single common origin in marsupials and eutherians, which last shared a common ancestor at least 148 million years (MY) ago, making marsupials an important comparative contrast to pregnancy in eutherians [3–5].

The comparison between pregnant and non-pregnant samples was chosen to identify the most differentially transcribed uterine genes during pregnancy. All the P and N samples used in this study came from opossums that had successfully given birth to at least one litter prior to this study. Based on what genes were being transcribed and the transcript number, the P transcriptomes were distinctly different from those of non-pregnant animals, whether N or V (Fig 1).

Sets of pregnancy-associated genes were identified as those at the intersection of differentially transcribed in the P versus all non-pregnant animals (N and V). Identifying the overlap in differentially transcribed genes in P vs N, P vs V, and P vs V2 & V3 generated gene sets with of high confidence for being significantly increased or decreased during opossum pregnancy. It is noteworthy that there were more genes with decreased transcription in the opossum uterus than increased transcription during pregnancy (n = 550; the difference between Fig 4A and 4B).

The top 100 most differentially transcribed opossum genes in the P and N groups were compared to results from pregnant uterine transcriptome studies in the pig, skink, and seahorse [28–30]. The pattern of differentially expression during pregnancy in skink had the most in common with the results in *M*. *domestica*. This similarity is unlikely due to shared structural similarities between the opossum and skink since the skink (*C*. *ocellatus*) has an epitheliochorial chorioallantoic placenta [28, 31, 32]. Rather the similarity in transcriptomes is likely due to the skink study focusing on a pregnancy time point more similar to that used here in the opossum. The opossum P samples were from uteruses preceding parturition by only 12–24 hours and there was likely to be late-pregnancy and parturition-specific gene transcription represented in the datasets.

In contrast there was less overlap with the transcriptome in the pig. This may due to the pig study focusing primarily on early pregnancy stages and also the pig’s chorioallantoic epitheliochorial placenta type and the source of embryonic nutrition, which primarily histotrophic rather than hemotrophic [29, 33, 34]. Likewise, there was very little overlap with the seahorse, which may be due to evolutionary distance or, again, the pregnancy time points examined.

Since there was substantial overlap in genes differentially transcribed in both the opossum and the skink, we focused on this comparison further. *Keratin 18* (*KRT18*) is has been previously shown to be essential to maintaining pregnancy in mice [35] and *KRT18* is increased during pregnancy in both opossum and skink (Table 4). *KRT18* is the only keratin gene that is increased in pregnant opossum (S2 Table). This is in contrast to eutherian pregnancy, such as in pigs and humans, where in addition to *KRT18*, *KRT8* and *KRT19* are also up-regulated [29, 36]. Other notable genes transcripts encoding structural molecules that are more abundant consistently in opossum and skink pregnancy are *fibronectin* (*FN1*) and *fibulin* (*FBLN1*) (Table 4). Both of these have been shown to be important to maintaining pregnancy in eutherian species such as mice [37, 38] and this conservation across a long evolutionary history is consistent with such proteins being critical to placental formation. In contrast *serpin peptidase inhibitor clade member 2* (*SERPINE2*), an up-regulated tissue remodeling gene in human, mouse, pig, and even seahorse pregnancy [29, 30, 39, 40], was among the most decreased gene transcripts during opossum pregnancy (Table 5). *SERPINE2* is also not differentially regulated in skinks [28]. Therefore, although *SERPINE2* transcription in pregnancy is shared between a teleost and eutherians, this does not appear to be a critically conserved gene.

Not surprisingly, an area of broad conservation was hormonal regulation. For example, *progesterone receptor* (*PGR*) had fewer transcripts during pregnancy in both opossum and skink (Table 5), and down-regulation of *PGR* in endometrial cells is observed in eutherian species at implantation [41–44]. Kin and colleagues used immunohistochemistry (IHC) to demonstrate that term pregnant *M*. *domestica* uterine epithelium and uterine glands express PGR weakly in comparison to non-pregnant uterine tissues [24]. The transcriptome results described here are consistent with that finding (S4 Table). *Oxytocin receptor* (*OXTR*) is more abundant in opossum at terminal pregnancy and in human pregnancy *OXTR* expression increases over time peaking prior to parturition and playing a role in inducing contractions [45–48]. Interestingly, Yamashita and Kitano [49] hypothesized that the evolution of *OXTR* associated with labor contractions in eutherians occurred after the marsupial-eutherian split. However, *OXTR* was a top 100 most abundant gene in pregnant opossum raising the possibility of its role in parturition being more ancient than the marsupial-eutherian split.

Prostaglandins have been shown to be important to normal birth mechanisms in other marsupials such as the tammar wallaby [50, 51]. Injection of exogenous prostaglandin F2 (PGF2) have also been shown to induce birthing in marsupials [50, 52]. The pregnant samples used in this study were taken from opossums that were within 24 hours of giving birth and it is probable that gene transcript values in these pregnant transcriptomes were influenced by birth mechanisms at work. Genes involved in prostaglandin synthesis and detection such as *prostaglandin E synthase* (*PTGES*), *prostaglandin-endoperoxide synthase 2* (*PTGS2*), and *prostaglandin E receptor 4 subtype EP4* (*PTGER4*) were all included in the increased in pregnancy-associated set (S2 Table).

Eight solute carrier proteins (*SLC39A9*, *SLC41A2*, *SLC16A5*, *SLC16A1*, *SLCO2A1*, *SLC39A8*, *SLC34A2L*, *SLC2A1*) are included in the top 100 most abundant genes in opossum during pregnancy (Table 4). This is consistent with ion transport being the most highly overrepresented GO term in the datasets. Two of these, *SLC39A9* and *SLC41A2* are zinc and magnesium ion transporters that are also up-regulated during skink pregnancy [28, 53, 54]. Transporters of cholesterol and chylomicrons like *ATP-binding cassette sub-family A member 2* (*ABCA2*) and apolipoprotein genes *APOA1*, *APOA4*, and *APOB* are also in the top 100 most abundant gene transcripts in opossum pregnancy (Table 4) which is consistent with what is known about cholesterol transport across eutherian placentas [55].

Two different non-pregnant control groups were used in this study due to concern over previous observations on sexual maturation in *M*. *domestica* [27]. Briefly, *M*. *domestica* females do not appear to reach reproductive maturity until they enter estrus and mate for the first time, a process dependent on male pheromones. Therefore, both virgin (V) females (pre-first estrus) as well as non-pregnant (N) experienced past breeders (post-first estrus) were used. In addition to providing controls for the pregnant state, this also allowed for a direct comparison between presumably immature pre-estrus and more mature post-estrus uterine tissues. In the analyses comparing the V group to P and N, only 12 genes were uniquely differentially transcribed in common (Table 3). Interestingly three of the four genes less abundant (*Ig J chain*, *Ig kappa chain*, *IgG heavy chain constant domain*) in virgin uterine tissue were genes associated with antibody expression (Table 3). This may indicate a need for humoral immunity in uterine tissues only after copulation and could point to a mechanism for controlling sexually transmitted infections in opossums. The morphological changes described by Stonerook and Harder [27], which is primarily organ weight, do not appear to be substantially reflected in changes in gene regulation in the uterus. *Actin alpha cardiac muscle 1* (*ACTC1*) and *keratin 13* (*KRT13*) are two of eight significantly more abundant genes in the virgin samples but no other major structural genes are differentially transcribed between the non-pregnant groups (Table 3).

The V group had the most variation between biological replicates than any other group as illustrated by V1 being an outlier (Fig 1). Nonetheless, V1 was transcriptomically more similar to the non-pregnant samples than to the pregnant ones. V1’s outlier status may have been due to her being 4.8 months and old at the time of collection, whereas the other two virgin animals (V2 and V3) were 10 months old. *M*. *domestica* individuals are generally considered to be sexually mature at 5–6 months old [56], but V1’s uterus was likely in a different stage of development from the older virgin animals. Aside from V1, all biological replicates were more similar to each other than to samples from other treatment groups (Fig 1). Non-pregnant individuals (groups N and V) were more similar to each other than they were to the pregnant individuals (Fig 1). This was an expected result and provided justification for retaining V1 in the analyses of differential transcription. There were more differentially transcribed genes shared between the P vs N and P vs V2 & V3 comparisons than between the P vs V and P vs V2 & V3 comparisons (Fig 4). This confirmed that V1 was skewing the differential transcription results in the P vs V comparison.

Kin and colleagues identified TFs spatially in *M*. *domestica* endometrium tissue sections in non-pregnant, pre-implantation pregnancy, and term pregnancy using IHC [24]. They concluded that the stromal mesenchymal cells seen in *M*. *domestica* endometrium are homologous to eutherian ESFs based on the expression of TFs and certain cytoskeletal proteins, and no marsupial has definitive decidualization. Two of the transcription factors (TFs) examined by Kin and colleagues were found in our pregnancy-associated gene sets. These were PGR, which was less abundant in pregnant samples, and CEBPB which was more abundant in pregnant samples (S4 Table). All samples examined in this study did have gene transcripts from the TFs evaluated in the Kin et al study (S4 Table). This is not surprising since Kin and colleagues observed transcription factors in a spatial manner by comparing IHC-stained endometrium of pregnant and non-pregnant *M*. *domestica*, whereas our study used whole sections of tissue without separating by cell type. Indeed, a limitation of the data sets used in this study is that they contain multiple cell and tissue types and therefore a specific transcript cannot be ascribed to a particular cell or tissue. Therefore in future studies on the genes identified as pregnancy-associated here, a spatial analysis of gene expression would be informative.

In conclusion, the analysis of the opossum late pregnancy uterine transcriptome enabled the discovery of a set of genes that can be described as being pregnancy-associated in the opossum. Curiously, morphological changes associated with sexual maturation in the opossum uterus reported previously are not obviously reflected in the transcriptome of this species. These analyses also revealed significant conservation of gene regulation among distantly related species. Reptiles and mammals are separated by over 300 MY of evolution and the opossum and skink do not share a common viviparous ancestor [57, 58]. While the similar nature of the gene regulation is likely indicative of convergence on common mechanisms in the evolution of live birth, the transcriptomic similarities in these studies could be explained by the similar pregnancy stages examined here and in the skink.

## Materials and Methods

### Animals and tissue collection

This study was approved under protocol numbers 13-100920-MCC and 15-200334-B-MC from the University of New Mexico Institutional Animal Care and Use Committee. Animals were from a captive-bred research colony housed at the University of New Mexico Department of Biology Animal Research Facility. Founders were obtained from *M*. *domestica* Populations 1 and 2 at the Southwest Foundation for Biomedical Research, San Antonio, TX [59]. All animals were fed *ad libitum* a diet of Labdiet Short Tailed Opossum #2 pellets (90%, Lab Supply TX) and dried mealworms (10%, Lab Supply TX). They were given drinking water *ad libitum* in sterile glass sipper bottles. All animals were housed individually in standard rat caging and bedding. When not breeding adult females and males were caged separately. All animals used in tissue collection were euthanized by inhaled isoflurane overdose (>5%) until breathing stopped for one minute.

Uterine tissues were collected from virgin (V), pregnant (P), and non-pregnant (N) experienced breeders. Non-pregnant past breeders had at least one successful pregnancy prior to harvesting. Uterine horns were excised and separated from surrounding tissues. In the case of pregnant uteri, the uterine horns were opened laterally and the intact embryos and amnions removed. Endometrium was separated from myometrium by teasing apart the tissue layers in shallow petri dishes filled with RNALater buffer (Ambion). In pregnant samples invasive fetal chorion and yolk sac membranes could not be fully separated from maternal endometrium. RNA extractions were either performed immediately or tissues were stored in RNALater overnight at 4°C. For longer-term storage, excess RNALater was removed and tissues were stored at -80°C until use.

### RNA extraction

Total RNA was isolated by homogenizing tissue in TRIzol (Ambion) using a sterile glass homogenizer followed by chloroform extraction and centrifugation at 4°C for 15 min. Protein and DNA contamination was removed using the PureLink RNA Mini (Ambion) and On-Column DNase Treatment (Invitrogen) kits following manufacturers' recommended protocols. RNA was stored in RNase-free water at -80°C. RNA quality was assessed using a 2100 Bioanalyzer (Agilent) and concentration was determined using a Qubit 2.0 Fluorometer (Life Technologies).

### cDNA library synthesis and Illumina sequencing

All cDNA library synthesis for high-throughput RNA-seq and Illumina sequencing procedures were performed at the National Center for Genome Resources (NCGR) in Santa Fe, New Mexico, USA. A Sciclone G3 Automated Liquid Handling Workstation (Caliper Life Sciences) with a Multi TEC Control for heating and cooling steps (INHECO) was used for the majority of liquid handling. Poly-A RNA was isolated using magnetic RNA Purification Beads (Dynabeads: Invitrogen). RNA was fragmented by high temperature (95°C for 8 min). SuperScript II enzyme (Intvitrogen), First Strand Master Mix (Illumina), and random hexamer primers (Illumina) were added to RNA to conduct first strand cDNA synthesis by reverse transcription. Second Strand Master Mix (Illumina) was used to conduct second strand cDNA synthesis, resulting in double-stranded cDNA. Double-strand cDNA was isolated using Agencourt AMPure XP beads (Beckman Coulter). Blunt end cDNA was prepared by removing 3’ overhangs and filling in 5’ overhangs using End Repair Mix (Illumina). 3’ ends were then adendylated using A-Tailing Mix (Illumina). Universal and barcoded TruSeq Adapters (Illumina) were ligated to cDNA ends. Polymerase Chain Reaction (PCR) was conducted with KAPA HiFi HotStart ReadyMix (KAPA Biosystems) and PCR Primer Cocktail (Illumina) to selectively amplify the cDNA fragments that had TruSeq adapters ligated to both ends. PCR products were purified using Agencourt AMPure XP beads. PCR product quality was assessed using a 2100 Bioanalyzer and cDNA quantity was measured by Nanodrop ND-1000 (Thermo Scientific). Samples were normalized to 10nM equimolar concentrations and pooled prior to flow cell injection. All Illumina sequencing was performed on an Illumina HiSeq 2000 instrument (Illumina) to generate 50bp paired-end reads. Read data was de-multiplexed (segregated by library index) using Illumina CASAVA v1.8.2 to produce FASTQ files. Thereafter, NCGR’s custom contaminant filtering pipeline removed anomalous sequences (i.e. Illumina PhiX control, library adapters, primer dimmers, and library indexes not part of the experiment). Quality assessment of read data was performed using FASTQC.

### Data access

All high-throughput sequence data sets generated for this study have been deposited at NCBI Sequence Read Archive (SRA) under the accession numbers SRR2969483, SRR2969536, SRR2970443, SRR2972728, SRR2972729, SRR2972792, SRR2972837, SRR2972840, and SRR2972848.

### Illumina read quality control, alignment, and quantification

All alignment and differential expression bioinformatics programs were run through the online Lumenogix platform (api.lumenogix.com, [60]). Reads were aligned to the annotated *M*. *domestica* genome (assembly: monDom5, annotation: Ensembl release 76) using Tophat2 [61, 62]. For a summary of Illumina read counts for this study see S1 Table. Read-count based differential expression was performed by Cufflinks, which normalizes by transcript length and apportions multi-mapped reads, and HTSeq which counts at the gene level and counts only uniquely mapped reads. Differential expression analysis was performed in three different algorithms: Cuffdiff, DESeq, and edgeR. Filtering was performed using log2-fold changes and adjusted p-value ≤ 0.05.

### Quantitative PCR

All RNA used for qPCR originated from the same RNA samples used for Illumina sequencing. Prior to cDNA synthesis, all RNA used in cDNA synthesis was treated with DNase using the TURBO DNA-free Kit (Ambion) according to manufacturers’ recommended protocols. 500ng of total RNA was used to make cDNA libraries by reverse transcriptase PCR (RT-PCR) using the SuperScript III First Strand Synthesis kit (Invitrogen) according to the manufacturer’s instructions. All cDNA libraries were made in triple replicates and then pooled to reduce bias generated during reverse transcription. Transcription levels of specific genes were assessed by qPCR using ABsolute Blue QPCR SYBR Green ROX Mix (Thermo Scientific) according to manufacturer’s instructions. Primers used in qPCR were designed for the *M*. *domestica* genome and according to manufacturer’s recommendations for qPCR primer properties. Primer sequences and properties are in S2 Table. All qPCR reactions were performed in triplicate repeats on an ABI Prism 7000 Sequence Detection System (Applied Biosystems) using the default cycling parameters with a dissociation step.

qPCR data were assessed by the Vandesompele method [63, 64] using *tyrosine 3-monooxygenase/tryptophan 5-monooxygenase activation protein zeta polypeptide* (*YWHAZ*) and *TATA box-binding protein* (*TBP*) as reference genes. These reference genes were chosen based on relatively consistent transcription across Illumina data samples (S5 Table), as well as literature recommending them as reference genes for placental gene expression studies [65, 66]. All pregnant and virgin qPCR gene transcription levels described here were relative to the non-pregnant past breeder group, and greater than 2-fold change was considered significant. Genes were chosen for qPCR confirmation based on being differentially transcribed in the Illumina data set, having >20 reads per sample in the Illumina data set, and novel qPCR primers passing efficiency testing. All primers were generated using the online tool Primer 3 (http://primer3.ut.ee/, [67]) with *M*. *domestica* gene sequences from the MonDom5 genome assembly. Primer sets used for qPCR and their associated properties are listed in S5 Table.

### Differential transcription analysis

Three separate differential expression analysis tools, Cuffdiff version 2.2.1, [68, 69], DESeq version 1.2.1 [70], and edgeR version 2.0.5 [71], were used to assess relative gene transcription between the P, N and V groups. All Venn diagrams were created using the R package VennDiagram [72]. The R package cummeRbund [73] was used to produce a dendrogram describing Jensen-Shannon (JS) distances between sample transcriptomes. The dendrogram was modified for readability in Microsoft PowerPoint. Since DESeq and edgeR use read counts and Cuffdiff uses Fragments Per Kilobase of transcript per Million mapped reads (FPKM) to calculate differential expression, the log2-fold change and associated adjusted p-value of genes were reported in cases where differential expression analysis results of specific genes were compared. The adjusted p-value threshold was ≤ 0.05 for all used differential expression programs.

### Gene Ontology analysis

Gene Ontology (GO) analysis was performed using online PANTHER tools (http://www.pantherdb.org/ [74, 75]. Specific gene sets were tested for statistical overrepresentation or underrepresentation of PANTHER GO-Slim Biological Process terms. Gene sets were compared to the whole uterine transcriptome gene set (all genes with expression of ≥ 1 FPKM in at least one uterine sample transcriptome) and GO terms with p < 0.05 (Bonferroni corrected for multiple testing) were considered significant. The “Unclassified” GO category was ignored in all analyses.

## Supporting Information

S1 Fig Gene transcription levels in P vs V according to differential expression programs compared to qPCR data. Log2-fold changes in the expression of membrane cofactor protein (CD46), MAC-inhibitory protein (CD59), interleukin 6 (IL-6), lysosomal-associated membrane protein 1 (LAMP1), and lumican (LUM) in the virgin group compared to the non-pregnant past breeder group. Log2-fold changes were according to Cuffdiff (pink bars), DESeq (yellow bars), edgeR (blue bars), and qPCR using the Vandesompele method (black bars). Red line indicates the threshold of log2-fold change needed for significance according to the Vandesompele method of relative quantification of qPCR data. None significant.
(TIF)

S1 Table Summary of Illumina read counts for each sequenced sample. (XLSX)

S2 Table Genes with significantly higher transcription in pregnant samples. List of all pregnancy-associated increased transcription genes (n = 932) in P group compared to N, V, and V2 & V3. Gene list includes transcript counts for all samples and p-values and log2-fold change values as measured by Cuffdiff, edgeR, and DESeq for all comparisons.
(XLSX)

S3 Table Genes with significantly lower transcription in pregnant samples. List of all pregnancy-associated decreased transcription genes (n = 1482) in P group compared to N, V, and V2 & V3. Gene list includes transcript counts for all samples and p-values and log2-fold change values as measured by Cuffdiff, edgeR, and DESeq for all comparisons.
(XLSX)

S4 Table Illumina read counts for selected transcription factor genes. (XLSX)

S5 Table Primers used in qPCR reactions and their properties. (XLSX)
