# Supplementary figures and images for: Transcriptomic Changes Associated with Pregnancy in a Marsupial, the Gray Short-Tailed Opossum Monodelphis domestica
Source: PLoS One. 2016 Sep 6;11(9):e0161608. doi: 10.1371/journal.pone.0161608 (PMC5012577; doi:10.1371/journal.pone.0161608)

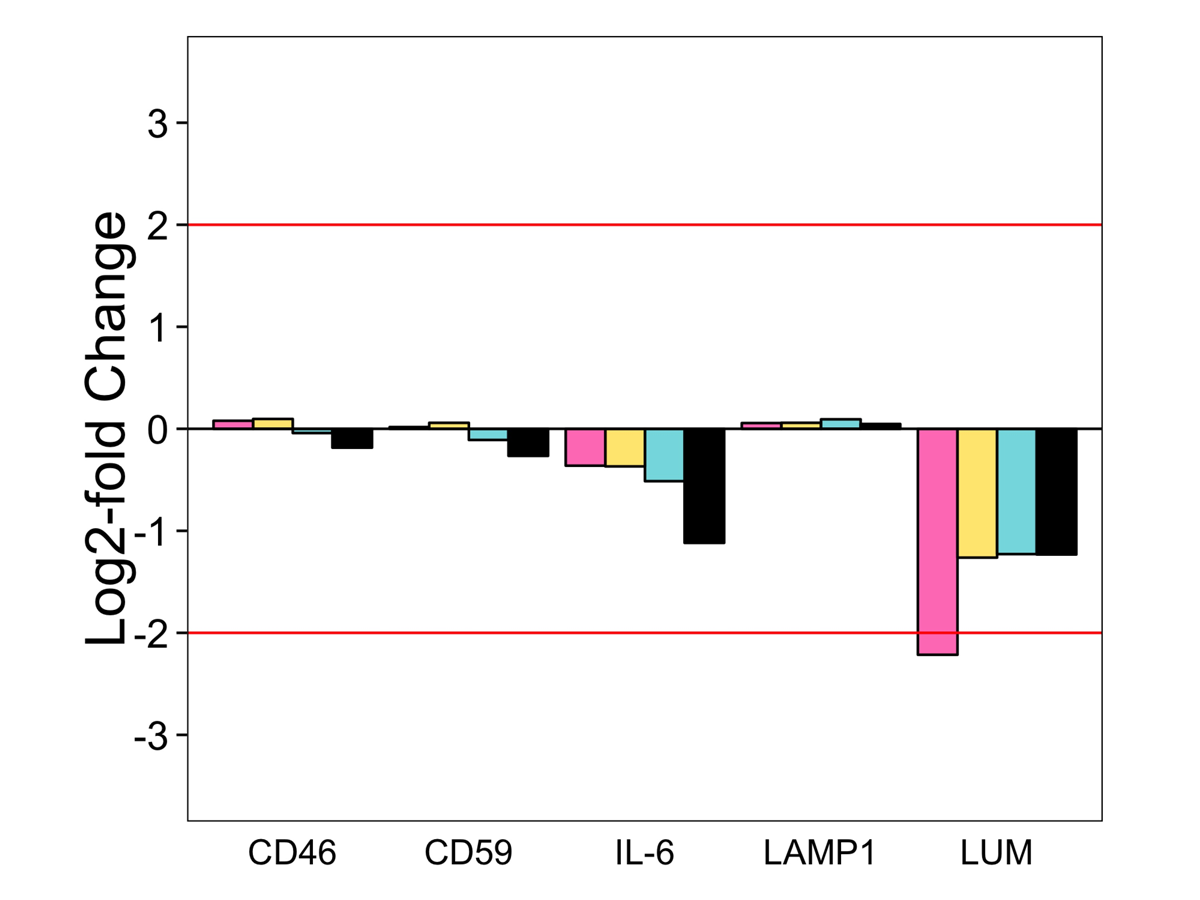

Supplement: S1 Fig — Log2-fold changes in the expression of membrane cofactor protein (CD46), MAC-inhibitory protein (CD59), interleukin 6 (IL-6), lysosomal-associated membrane protein 1 (LAMP1), and lumican (LUM) in the virgin group compared to the non-pregnant past breeder group. Log2-fold changes were according to Cuffdiff (pink bars), DESeq (yellow bars), edgeR (blue bars), and qPCR using the Vandesompele method (black bars). Red line indicates the threshold of log2-fold change needed for significance according to the Vandesompele method of relative quantification of qPCR data. None significant. (TIF) [file pone.0161608.s001.tif]
